# Nitrofurantoin failure in males with an uncomplicated urinary tract infection: a primary care observational cohort study

**DOI:** 10.3399/BJGP.2022.0354

**Published:** 2023-02-21

**Authors:** Tamara N Platteel, Marijn T Beets, Hendrik A Teeuwissen, Thijs ten Doesschate, Janneke HHM van de Wijgert, Roderick P Venekamp, Alma C van de Pol

**Affiliations:** Julius Center for Health Sciences and Primary Care, University Medical Center Utrecht, Utrecht University, Utrecht, Netherlands.; Julius Center for Health Sciences and Primary Care, University Medical Center Utrecht, Utrecht University, Utrecht, Netherlands.; Julius Center for Health Sciences and Primary Care, University Medical Center Utrecht, Utrecht University, Utrecht, Netherlands.; Julius Center for Health Sciences and Primary Care, University Medical Center Utrecht, Utrecht University, Utrecht; Department of Internal Medicine, Jeroen Bosch Hospital, ‘s-Hertogenbosch, Netherlands.; Julius Center for Health Sciences and Primary Care, University Medical Center Utrecht, Utrecht University, Utrecht, Netherlands.; Julius Center for Health Sciences and Primary Care, University Medical Center Utrecht, Utrecht University, Utrecht, Netherlands.; Julius Center for Health Sciences and Primary Care, University Medical Center Utrecht, Utrecht University, Utrecht, Netherlands.

**Keywords:** general practice, nitrofurantoin, treatment failure, urinary tract infection

## Abstract

**Background:**

Nitrofurantoin is the first-choice antibiotic treatment for uncomplicated urinary tract infections (UTIs) in males according to the Dutch primary care UTI guideline. However, prostate involvement may be undetected and renders this treatment less suitable.

**Aim:**

To compare the nitrofurantoin failure fraction with that found with use of other antibiotics in adult males diagnosed by their GP with an uncomplicated UTI, as well as GP adherence to the Dutch primary care UTI guideline.

**Design and setting:**

Retrospective observational cohort study using routine healthcare data for males seeking care at GP practices participating in the Julius GP Network from 2014 to 2020.

**Method:**

Medical records were screened for signs and symptoms of complicated UTIs, antibiotic prescriptions, and referrals. Treatment failure was defined as prescription of a different antibiotic within 30 days after initiation of antibiotic therapy and/or acute hospital referral. The effects of age and comorbidities on failure were assessed using multivariable logistic regression.

**Results:**

Most UTI episodes in males were uncomplicated (*n* = 6805/10 055 episodes, 68%). Nitrofurantoin  was prescribed in 3788 (56%) of uncomplicated UTIs, followed by ciprofloxacin (*n* = 1887,  28%), amoxicillin/clavulanic acid (*n* = 470,  7%), and trimethoprim/sulfamethoxazole (*n* = 285, 4%). Antibiotic failure occurred in  25% (95% confidence interval [CI] = 23 to 26), 10% (95% CI = 9 to 12), 20% (95% CI = 16 to 24), and 14% (95% CI = 10 to 19) of episodes, respectively. The nitrofurantoin failure fraction increased with age. Comorbidities, adjusted for age, were not associated with nitrofurantoin failure.

**Conclusion:**

Nitrofurantoin failure was common in males with uncomplicated UTI and increased with age.

## INTRODUCTION

Uncomplicated urinary tract infection (UTI), also known as cystitis, afebrile UTI, or UTI without tissue invasion, is typically caused by invasion of gut or skin bacteria, or overgrowth of resident bacteria, in the urinary tract. Internationally, there is ongoing debate about whether males can actually have an uncomplicated UTI. Some experts argue that the prostate is always involved to some extent indicating a complicated UTI, but evidence to support this is lacking. In some countries, such as the UK, Sweden, and the Netherlands, the distinction between complicated and uncomplicated UTIs in males is made in the national guidelines, although definitions differ slightly across guidelines.^1^^–^^3^ In the Dutch primary care UTI guideline, an uncomplicated UTI is defined as a UTI without signs of systemic inflammation, that is, absence of fever, malaise, cold shivers, flank or perineal pain, signs of sepsis, or delirium.^2^

Although UTIs are much less common in males than in females, the incidence increases considerably with age. The annual incidence rises from 11 per 1000 person–years in males aged <65 years to 174 per 1000 person–years in males aged  ≥85 years, corresponding to around 165 000 males with UTIs in the Netherlands annually.^2^

Evidence for optimal treatment of uncomplicated UTIs in males is scarce. The Dutch primary care UTI guideline recommends nitrofurantoin 100 mg slow release twice daily for 7 days as the first-choice antibiotic for males with an uncomplicated UTI.^2^ This is mainly based on expert opinion, extrapolation from scientific evidence related to uncomplicated UTIs in females, and low *Escherichia coli* (*E. coli*) nitrofurantoin resistance rates in the Netherlands.^2^ However, a higher risk of nitrofurantoin treatment failure in males than in females could be anticipated for two main reasons: first, males with uncomplicated UTIs may have undetected involvement of the prostate and nitrofurantoin hardly reaches therapeutic concentrations in the prostate;^4^^,^^5^ and second, although most UTIs in females are caused by *E.coli*, UTIs in  males  are  often  caused  by  non-*E.coli* uropathogens. A study from the Netherlands showed that approximately half of UTI episodes in males are caused by *E. coli*, with *Klebsiella* spp found in 6%, *Proteus mirabilis* in 5%, *Enterococcus* spp in 5%, other  Gram-negatives  in  11%,  and  other  Gram-positives in 12%.^6^ Nitrofurantoin is less effective against non-*E. coli* uropathogens, (that is, *Proteus* spp, *Pseudomonas* spp, and *Serratia* spp have intrinsic resistance to nitrofurantoin). GPs may therefore intuitively deviate from current Dutch guideline recommendations and prescribe other antibiotics such as the second-choice antibiotic trimethoprim, or antibiotics that are only recommended for complicated UTIs: ciprofloxacin, amoxicillin/clavulanic acid, and trimethoprim/sulfamethoxazole. However, antimicrobial resistance rates for *E. coli* in males in Dutch primary care are 22% for trimethoprim, 17% for ciprofloxacin, 33% for amoxicillin/clavulanic acid, and 20% for trimethoprim/sulfamethoxazole.^2^ Furthermore, ciprofloxacin use is associated with a risk of serious adverse events.^7^ Nitrofurantoin treatment causes less resistance than these other antibiotics and has a favourable safety profile.

**Table:** How this fits in

Nitrofurantoin is the first-choice antibiotic for treatment of uncomplicated urinary tract infections (UTIs) in males in the Dutch primary care UTI guideline. Nonetheless, the risk of nitrofurantoin failure in this condition is largely unknown. This study reports the failure fractions of nitrofurantoin compared with other commonly prescribed antibiotic treatments for uncomplicated UTIs in males. Antibiotic failure occurred in 25% of nitrofurantoin-treated episodes, increasing with age; the figures were 10% for ciprofloxacin, 20% for amoxicillin/clavulanic acid, and 14% for trimethoprim/sulfamethoxazole, not increasing with age. These results will help to inform treatment decisions regarding uncomplicated UTI in males.

The aims of this study were to inform the Dutch primary care UTI guideline by comparing the failure fractions of the various commonly GP-prescribed antibiotics in uncomplicated UTIs in males, and to assess GP adherence to the current Dutch guideline.

## METHOD

### Design and study population

This retrospective observational cohort study was conducted using anonymised routine healthcare data from the Julius General Practitioners’ Network (JGPN) collected from January 2014 to December 2020. The network covers 84 general practices providing care during office hours in Utrecht and surrounding areas. Participating practices and their registered patients (approximately 370 000 individuals) are representative of the Dutch population as a whole.^8^ The study population consisted of adult males (aged ≥18 years) with uncomplicated UTI, as diagnosed by their GP,  who  received  a  UTI-related  antibiotic  prescription for that UTI episode. Patients were able to contribute multiple UTI episodes to the dataset.

### Outcome data collection and definitions

GPs participating in the JGPN register a diagnosis for each consultation using International Classification of Primary Care (ICPC) codes. As a first step, medical record data were collected from all consultations of adult males with ICPC codes U01 (dysuria/painful urination), U02 (urinary frequency/urgency), U70 (pyelonephritis/pyelitis), U71 (cystitis), and Y73 (prostatitis) for which a UTI-related antibiotic (nitrofurantoin, trimethoprim, ciprofloxacin, amoxicillin/clavulanic acid, trimethoprim/sulfamethoxazole, fosfomycin, or norfloxacin) was prescribed within 7 days before and after the start date. A UTI episode after a UTI consultation-free interval of 30 days was considered a new UTI episode. Second, UTI episodes were excluded if:

- the patient had an indwelling urinary catheter;
- no information on patient-reported symptoms and GP-assessed signs was available;
- one UTI episode was associated with two prescriptions for different antibiotics on the same day;
- the antibiotic prescription was for prophylactic or future use, and
- the patient was anatomically female.

Next, medical records of all remaining UTI episodes were manually screened and scored for patient-reported symptoms and GP-assessed signs of a complicated UTI, namely fever reported by patients and/or assessed  by  GPs,  patient-reported  malaise  and/or cold shivers, and GP-assessed ‘clinically severely ill’, costovertebral angle tenderness, perineal pain, and/or signs of delirium.^2^ A UTI was considered uncomplicated when all of these signs and symptoms were absent. If a sign or symptom was not recorded it was considered absent. Screening and scoring of 400 variables regarding signs and symptoms of complicated UTI was done in duplicate by two independent investigators with an agreement of 99% (kappa 0.94 [almost perfect agreement]).

The following data were extracted for each uncomplicated UTI episode: age, comorbidities (diabetes, cardiovascular, pulmonary, oncological, nephrological, urological, and neurological and immunocompromising conditions; see Supplementary Appendix S1), number and type of antibiotic prescriptions using Anatomical Therapeutic Chemical codes, and hospital referrals.

Treatment failure was defined as an antibiotic prescription for a different antibiotic >1 day after the initial prescription or an acute hospital referral to urology or internal medicine >1 day after the antibiotic prescription.

The Dutch UTI guideline recommends nitrofurantoin (first choice) or trimethoprim (second choice) for treatment of uncomplicated UTI in males.^2^ GPs were therefore considered adherent to the guideline if they prescribed one of those antibiotics. If treatment failure had occurred in the year before the current UTI episode, ciprofloxacin, amoxicillin/clavulanic acid, and trimethoprim/sulfamethoxazole were considered adherent as well.

As data on UTI episodes from 2013 were not available, treatment failure in the year before 2014 could not be determined. The year 2014 was therefore excluded from the adherence analysis.

### Analyses

Uncomplicated UTI incidence rates in males were calculated by dividing the number of UTI episodes by the number of person–years available in the JGPN database, overall and stratified by age group. Baseline characteristics were described descriptively. Treatment failure fractions for the various antibiotic regimens were expressed as percentages with 95% confidence intervals (CIs). Only the first course of antibiotics given for a UTI episode was included to determine ‘prescribed antibiotic’ statistics. Guideline adherence by GPs was calculated by dividing the number of uncomplicated UTI episodes in which the GP prescribed an antibiotic according to the guideline recommendation by the total number of uncomplicated UTI episodes. A secondary analysis was performed to assess GPs’ adherence with the recommended nitrofurantoin or trimethoprim treatment duration of ≥7 days.

A random intercept model was performed to determine the effect of age and comorbidities on nitrofurantoin failure. Statistical analyses were performed with MedCalc Version 20.110 and IBM SPSS Statistics (version 26.0.0.1).^9^

## RESULTS

### Study population

**Figure 1.**
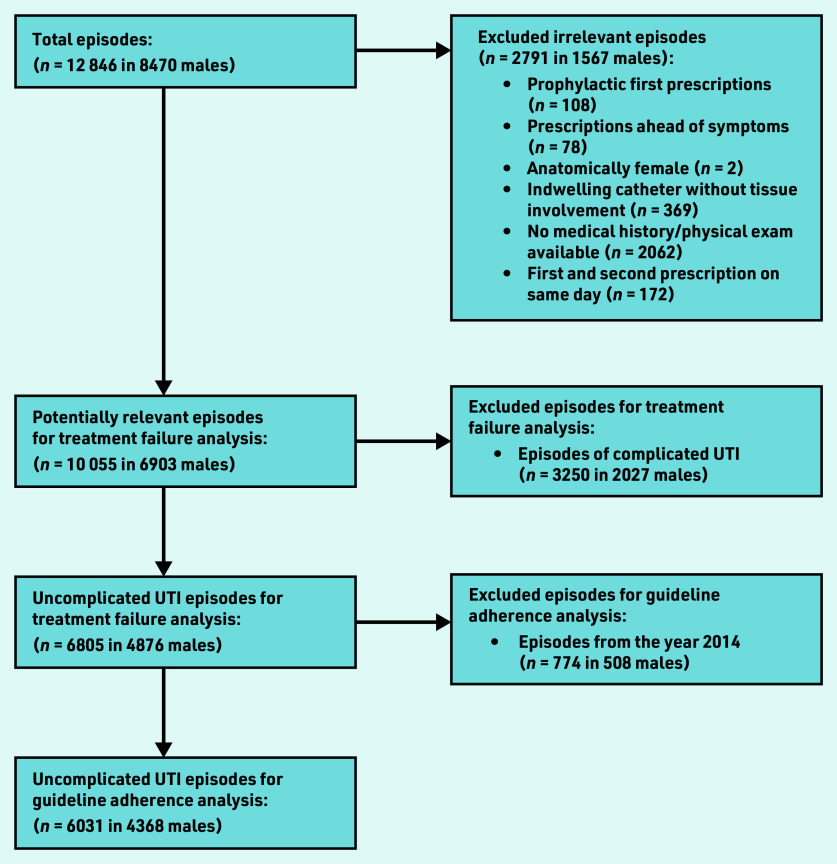
*Flowchart of included episodes. UTI = urinary tract infection.*

Between 2014 and 2020, an annual mean of 150 450 adult males were registered in the JGPN database. These males experienced 12 846 UTI episodes, of which 2791 episodes (22%) were excluded for various reasons, leaving 10 055 episodes in 6903 individual males suitable for inclusion (Figure 1).

### Incidence and treatment of uncomplicated UTI in males

Of the 10 055 episodes, 6805 episodes (68%) in 4876 individual males were defined as uncomplicated UTI episodes. The baseline characteristics associated with these 6805 episodes are shown in Table 1.

**Table 1.** Baseline characteristics of the study population

**Characteristic**	**Uncomplicated UTI**
**Total episodes,^a^** ***n***	6805

**Age, median (IQR)**	69 (55–79)

**Age groups, years,** ***n* (%)**	
18–39	727 (11)
40–64	1972 (29)
65–79	2425 (36)
≥80	1681 (25)

**Comorbidities,** ***n* (%)**	
COPD/asthma	1097 (16)
Diabetes	1600 (24)
Cardiovascular disease	2586 (38)
Oncological disease	1082 (16)
Neurological disease	109 (2)
Nephrological disease	506 (7)
Urological disease	115 (2)
Immunocompromised	472 (7)

a *A total of 6805 episodes in 4876 individual males. COPD = chronic obstructive pulmonary disease. IQR = interquartile range. UTI = urinary tract infection.*

The overall incidence of uncomplicated UTI was 6.5/1000 person–years. The incidence increased with age: (18–39 years: 1.7/1000  person–years; 40–64 years: 4.5/1000 person–years; 65–79 years 18/1000 person–years  and  >80 years: 45/1000 person–years)(data not shown).

The most commonly prescribed antibiotic for uncomplicated UTIs in males was nitrofurantoin (*n* = 3788, 56%), followed by ciprofloxacin (*n* = 1887, 28%), amoxicillin/clavulanic acid (*n* = 470, 7%), trimethoprim/sulfamethoxazole (*n* = 285, 4%), and other antibiotics (trimethoprim, fosfomycin, and norfloxacin combined: *n* = 375, 6%) (Table 2). The second-choice antibiotic trimethoprim was only prescribed in 185 episodes.

**Table 2.** Treatment failure for uncomplicated UTIs in males by antibiotic regimen

**Antibiotic**	**Episodes failed**		**Total prescriptions,*n* (% of total)**
	***n***	**% (95% CI)**	
Nitrofurantoin	929	25 (23 to 26)	3788 (56)
Ciprofloxacin	198	10 (9 to 12)	1887 (28)
Amoxicillin/clavulanic acid	92	20 (16 to 24)	470 (7)
Trimethoprim/sulfamethoxazole	40	14 (10 to 19)	285 (4)
Trimethoprim	46	25 (18 to 33)	185 (3)
Fosfomycin	26	22 (15 to 33)	116 (2)
Norfloxacin	14	19 (10 to 32)	74 (1)
Total	1345	20 (19 to 21)	6805

*UTI = urinary tract infection.*

Acute hospital referrals occurred in 2% (*n* = 87) of episodes treated with nitrofurantoin, 3% (*n* = 6) treated with trimethoprim, 3% (*n* = 62) treated with ciprofloxacin, 1% (*n* = 7) treated with amoxicillin/clavulanic acid, and 4% (*n* = 10) of episodes treated with trimethoprim/sulfamethoxazole (data not shown).

### Antibiotic failure in uncomplicated UTI in males

Nitrofurantoin failure occurred in 25% (95% CI = 23 to 26) of the nitrofurantoin-treated episodes (92% antibiotic switch, 5% referral, and 4% both switch and referral) (Table 2) (data not shown). The nitrofurantoin failure percentage increased with age (18–39 years: 13%, 40–64 years: 22%, 65–79 years: 27%, and >80 years: 29%; Supplementary Table S1).

Age (in years) was statistically significantly associated with a higher odds of nitrofurantoin failure (odds ratio [OR] 1.01, 95% CI = 1.01 to 1.02). Comorbidities, when adjusted for age, were not significantly associated with nitrofurantoin failure (see Supplementary Table S2).

The trimethoprim, ciprofloxacin, amoxicillin/clavulanic acid, and trimethoprim/sulfamethoxazole failure percentages were 25% (95% CI = 18 to 33, 87% switch, 7% referral, and 7% both), 10% (95% CI = 9 to 12; 79% switch, 19% referral, and 2% both), 20% (95% CI = 16 to 24; 93% switch, 5% referral, and 1% both), and 14% (95% CI = 10 to 19; 85% switch, 13% referral, and 3% both), respectively (Table 2). Age (in years) was not associated with a higher odds of trimethoprim, ciprofloxacin, amoxicillin/clavulanic acid, and trimethoprim/sulfamethoxazole failure.

### Guideline adherence in uncomplicated UTI in males

Data from 6031 episodes (in 4368 individual males) were available for assessing guideline adherence (Figure 1). In 3712 episodes (62%), the prescribed antibiotic was judged adherent to the Dutch primary care UTI guideline (Table 3). In the 2319 episodes  classified  as  non-adherent  to  the guideline, ciprofloxacin was prescribed most commonly (68%), followed by amoxicillin/clavulanic acid (16%) and trimethoprim/sulfamethoxazole (9%). Age was  not  associated  with  non-adherent  prescribing, but the presence of urological (OR 1.55, 95% CI = 1.29 to 1.87), nephrological (OR 2.01, 95% CI = 1.36 to 2.96) or immunocompromising (OR 1.23, 95% CI = 1.01 to 1.49) comorbidities were (data not shown).

**Table 3.** Results for guideline adherence in uncomplicated UTI episodes

**Category**	**Adherent, *n* (%)**	**Non-adherent, *n* (%)**	**Total episodes,*n***
**Antibiotic**			
Total	3712/6031 (62)	2319/6031 (39)	6031
Nitrofurantoin	3401/6031 (56)	0/6031 (0)	3401
Trimethoprim	168/6031 (3)	0/6031 (0)	168
Ciprofloxacin	80^a^/6031 (1)	1582/6031 (26)	1662
Amoxicillin/clavulanic acid	39^a^/6031 (1)	364/6031 (6)	403
Trimethoprim/sulfamethoxazole	28^a^/6031 (1)	214/6031 (4)	242
Fosfomycin	0/6031 (0)	109/6031 (2)	109
Norfloxacin	0/6031 (0)	46/6031 (1)	46

**Age groups, years**			
18–39	391/613 (64)	222/613 (36)	613
40–64	1031/1708 (60)	677/1708 (40)	1708
65–79	1364/2194 (62)	830/2194 (38)	2194
>80	930/1516 (61)	586/1516 (39)	1516

**Comorbidities**			
None	1206/1900 (64)	694/1900 (37)	1900
≥1	2510/4131 (61)	1621/4131 (39)	4131
Asthma/COPD	596/983 (61)	387/983 (39)	983
Diabetes	876/1448 (61)	572/1448 (40)	1448
Cardiovascular	1449/2346 (62)	897/2346 (38)	2346
Cancer	591/1006 (59)	415/1006 (41)	1006
Nephrological	250/483 (52)	233/483 (40)	483
Urological	47/105 (45)	58/105 (55)	105
Neurological	58/97 (60)	39/97 (40)	97
Immunocompromised	250/438 (57)	188/438 (43)	438

a *Considered adherent because of treatment failure in 365 days prior to the current UTI episode. COPD = chronic obstructive pulmonary disease. UTI = urinary tract infection.*

### Guideline adherence: duration of antibiotic treatment

Data on antibiotic treatment duration was available for 3358 nitrofurantoin and 164 trimethoprim prescriptions. Prescriptions adhered to the recommended length of 7 days for nitrofurantoin in 3052 of 3368 (91%) episodes and for trimethoprim in 133 of 164 (81%) episodes. Nitrofurantoin prescriptions for ≤5 days (*n* = 234) did not result in a higher treatment failure risk compared with a prescription for 7 days in males with an uncomplicated UTI (21% and 25%, respectively) (data not shown).

## DISCUSSION

### Summary

This observational cohort study reveals that nitrofurantoin, the first-choice treatment for uncomplicated UTI in males in the Netherlands, failed in 25% of episodes, which was higher than the failure percentages of ciprofloxacin, amoxicillin/clavulanic acid, and trimethoprim/sulfamethoxazole (10%, 20%, and 14%, respectively). The failure percentage of  trimethoprim,  the  second-choice  treatment, was also 25%, but the number of trimethoprim prescriptions was small. Nitrofurantoin failure significantly increased with age, ranging from 13% in those aged 18–39 years to 29% in those aged >80 years. GPs were shown to deviate from the Dutch UTI guideline in 38% of uncomplicated UTI episodes, mostly prescribing ciprofloxacin instead of nitrofurantoin or the infrequently prescribed second-choice trimethoprim.

### Strengths and limitations

Major strengths of the current study are the large sample size allowing robust age-specific subgroup analyses and the use of well-documented primary care electronic routine care data for 7 consecutive years. Using the medical text from UTI consultations it was possible to discern uncomplicated from complicated UTI episodes and to not only take antibiotic switches but also treatment failures leading to an acute hospital referral into account.

Some limitations deserve further discussion. First, as this was a medical record study, it was not possible to ascertain whether tissue invasion was definitely absent. Tissue involvement might have gone undetected by the GP. This is, however, no different than in actual routine care. Besides, not only ICPC codes for cystitis, but also for prostatitis and pyelonephritis were included to obtain consultation data for all types of UTIs. As free medical text entered by the GP is more reliable than ICPC coding, all signs and symptoms of tissue invasion were scored manually to distinguish uncomplicated from complicated UTIs. Episodes with ICPC codes for prostatitis and pyelonephritis accounted for 4% of all uncomplicated UTI episodes. Even if the inclusion of such episodes would have introduced some misclassification, it was anticipated this would be of minor influence on the findings.

Second, data from out-of-hours services, such as prescriptions and referrals, and deaths were not available, which might have led to an underestimation of the observed treatment-failure fractions. It is, however, unlikely that a potential bias would differ for the various UTI-related antibiotic prescriptions. Also, no data were available on intolerances, allergies, or interactions with other medications, which might justify some non-adherent prescriptions. Besides, GPs might have other good reasons to deviate from guideline recommendations, for example, initiating a more aggressive treatment in vulnerable patients with clinically uncomplicated UTIs.

Third, by classifying all re-prescriptions with a different antibiotic and all acute referrals to urology or internal medicine as a treatment failure, some overestimation of treatment failure might have occurred. Some re-prescriptions might have been issued for other reasons than treatment failure, such as adverse effects. Also, patients might have been referred to urology or internal medicine for another reason but, as the time window for referrals was short and the number of referrals low, the authors believe that the potential impact of such misclassification will be minor.

Fourth, this study is observational (that is, non-randomised) and confounding by indication may therefore be present: the groups of males treated with nitrofurantoin versus another antibiotic might not be comparable regarding disease severity and other factors contributing to treatment failure. This may, for example, explain why hospital referrals were less common in males treated with nitrofurantoin compared with males treated with ciprofloxacin. It seems likely that patients with more severe symptoms are prescribed ciprofloxacin more often than patients with milder complaints.

Fifth, in line with clinical practice, it was not know whether patients used their antibiotics as prescribed. Sixth, there is ongoing debate whether the effectiveness of nitrofurantoin is reduced in patients with a low estimated glomerular filtration rate (eGFR).^10^^,^^11^ According to the Dutch primary care UTI guideline, nitrofurantoin is contraindicated in patients with an eGFR <30 mL/min/1.73 m^2^^.^^2^ Unfortunately, information about patient’s actual eGFR was not available in the current study. As GPs as well as pharmacies receive an alert if nitrofurantoin is prescribed to a patient with an eGFR <30 mL/min/1.73 m^2^, the authors consider it unlikely that many patients would have had a eGRF <30 mL/min/1.73 m^2^. However, it cannot be excluded that nitrofurantoin failure was more likely in males with eGFR between 30 and 90 mL/min/1.73 m^2^. Further research into this potential association is warranted.

### Comparison with existing literature

The Netherlands is one of few countries worldwide recommending nitrofurantoin as a first-choice treatment for uncomplicated UTIs in males. Most available studies are therefore of Dutch origin. A previous smaller study from the same author group showed a similar nitrofurantoin failure percentage (27%) as this study.^5^ However, the current study adds valuable information. First, in the previous study, data on signs and symptoms were not available, making a distinction between complicated and uncomplicated UTIs impossible. Second, only nitrofurantoin prescription data from older males were available as opposed to all antibiotic types in all age groups in the current study, making comparisons between antibiotic regimens possible. Additionally, the Dutch primary care UTI guideline cites an unpublished study by the Netherlands Institute for Health Services estimating the nitrofurantoin failure percentage to be 27% in males of all ages.^2^ However, their treatment failure definition also included antibiotics that were likely not prescribed for UTIs, and the failure period was defined as 60 days. A third Dutch outpatient study showed that 5.4% of male patients treated with nitrofurantoin were prescribed a different antibiotic within 3 days across all ages.^12^ This much lower treatment failure risk may likely be explained by the 3-day treatment failure period as opposed to the 30-day period in the current study.

To the authors’ knowledge, only two studies on nitrofurantoin efficacy in males from outside the Netherlands have been published. A retrospective study in 485 American male veteran patients with lower UTIs showed a clinical cure fraction of 77%.^13^ However, 32% of the males had a catheter inserted at the time nitrofurantoin was prescribed, making it difficult to generalise the results to males with uncomplicated UTIs. Another retrospective study from Sweden in 69 males with a lower UTI showed nitrofurantoin failure in only one patient, but the sample size was very small.^14^

The 62% guideline adherence observed in the current study was comparable with the 50% adherence in the only other study, to the authors’ knowledge, that has been published about adherence to the guidelines for males.^15^ That study included 169 Dutch males with an uncomplicated UTI. Also comparable is the very low prescription fraction of 3% in the current study and 4.4% in Ganzeboom *et al* ^15^ for the second-choice trimethoprim. This might be related to the failure fraction of 25%, which is comparable with nitrofurantoin.

### Implications for research and practice

The current study shows higher rates of nitrofurantoin failure compared with ciprofloxacin, amoxicillin/clavulanic acid, and trimethoprim/sulfamethoxazole in males with uncomplicated UTI, especially in those of older age. As this is based on observational data causal inferences cannot be drawn and the implications for what the consequences are for clinical practice remains to be elucidated.

Based on the current findings, a randomised controlled trial comparing various antibiotic regimens is warranted to robustly determine the optimal treatment strategy for males with an uncomplicated UTI.

In conclusion, nitrofurantoin failure was common in males with an uncomplicated UTI and this increased with age. GPs deviated from the Dutch UTI guideline in 38% of the uncomplicated UTI episodes, mostly prescribing ciprofloxacin instead of nitrofurantoin.

## Data

Data are not publicly available due to ethical and legal restrictions.
